# Single Solitary Fibrous Tumor Brain Metastasis in a Patient with Simultaneous Adenocarcinoma of the Lung: Case Report and Review of the Literature

**DOI:** 10.1155/2020/3938270

**Published:** 2020-04-07

**Authors:** Erin A. Kaya, Jonathan D. Carlson, Cheddhi J. Thomas, Aaron E. Wagner, Robert K. Fairbanks, Wayne T. Lamoreaux, Christopher M. Lee

**Affiliations:** ^1^Department of Radiation Oncology, Cancer Care Northwest and Gamma Knife of Spokane, Spokane, WA, USA; ^2^Washington State University (WSU) Elson S. Floyd College of Medicine (ESFCOM), Spokane, WA, USA; ^3^Inland Neurosurgery and Spine Associates, Spokane, WA, USA; ^4^Incyte Diagnostics, Spokane, WA, USA

## Abstract

We present a unique case of a patient simultaneously diagnosed with solitary fibrous tumor (SFT) and unrelated adenocarcinoma of the lung, both proven with separate pathology. It was subsequently found that the SFT had metastasized to the brain by additional pathology, and not the predicted adenocarcinoma. SFTs are a rare mesenchymal neoplasm that accounts for less than 2% of all reported soft tissue tumors. SFTs most commonly arise in the thoracic cavity, but are frequently found in various locations throughout the body, and rarely metastasize to the brain. This case highlights that rare neoplasms, such as SFT, should not be ruled out as a potential cause of metastasis. Due to the rarity of this clinical situation, we also provide a review and discussion of previously reported SFT cases and the use of postoperative radiation therapy. The optimal treatment for individual patients remains unclear in this unique situation. Surgical resection followed by adjuvant Gamma Knife radiation therapy to the surgical bed appears to be a safe option for local treatment of SFT in select patients. Further studies are needed of this rare clinical situation in order to better understand and optimize future treatments for patients with SFT and metastasis to the brain.

## 1. Introduction

We present a unique case of a patient with simultaneously diagnosed solitary fibrous tumor of the lung and adenocarcinoma of the lung, and who was later found to have a single solitary fibrous tumor metastasis to the brain. This patient underwent surgical resection for the brain metastasis followed by Gamma Knife treatment to the surgical cavity. At this time, there are no other reported cases in the literature with this specific course of treatment for the single brain metastasis from solitary fibrous tumor pathology.

Solitary fibrous tumors (SFTs) are rare mesenchymal neoplasms that account for less than 2% of all reported soft tissue tumors [1]. SFTs are most commonly benign, but 10–20% of cases have been reported to be malignant. SFTs usually occur in middle-aged patients and equally in men and women [2]. The most common sites for metastasis are the lungs (61%), pleura (49%), liver (41%), bones (41%), and peritoneum (41%) [3]. SFT has a low reported incidence of metastasis to the brain, especially low when compared to lung adenocarcinoma [4].

## 2. Case Report

The patient is a 70-year-old male Navy veteran who served on the Mekong in Vietnam for 14 months, during which time he reports that he was exposed to Agent Orange. He has a previous history of congestive heart failure, coronary artery disease with coronary artery bypass grafting in 2015, and chronic obstructive pulmonary disease from tobacco.

**Figure 1:**
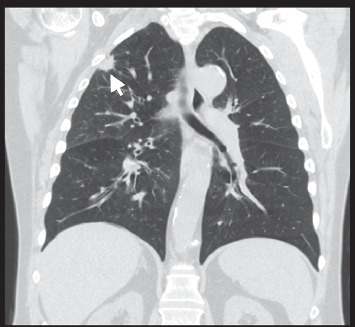
Noncontrast CT chest, coronal view. Visible is a 1.5 cm tumor mass in the right upper lobe of the lung.

**Figure 2:**
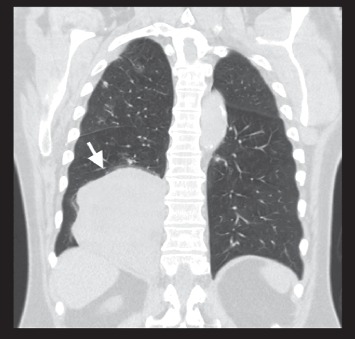
Noncontrast CT chest, coronal view. Visible is a 13.0 × 12.8 × 7.5 cm tumor mass in the right lower lobe of the lung.

**Figure 3:**
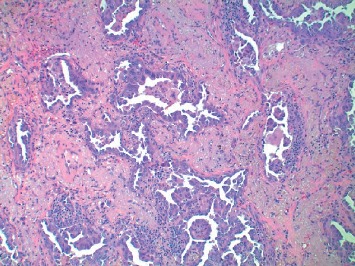
Hematoxylin and Eosin staining, low-power 100x view of adenocarcinoma. Tumor shows an acinar growth pattern.

He presented with progressive shortness of breath and underwent a workup with CT scan of the chest without contrast, which revealed two distinct appearing tumors, one in the right upper lobe with a tumor size of 1.5 cm and another in the right lower lung with a tumor size of 13 cm (see Figures 1 and 2). He underwent a right upper lobe wedge resection and a right lower lobectomy for these tumors. Pathology revealed two distinct neoplasms. The right upper lobe tumor was a moderately differentiated lung adenocarcinoma, stage pT2a (see Figure 3). Pathology revealed focal invasion of the visceral pleura, confirmed by elastic stain.

**Figure 4:**
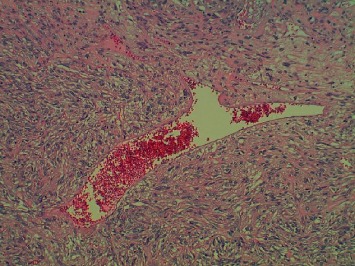
Hematoxylin and Eosin, low-power 100x view of solitary fibrous tumor. Mesenchymal neoplasm comprised by spindled cells embedded within a collagen matrix. The tumor is fed by a mixture of small slit-like vessels and large ectatic vessels with a characteristic “staghorn” appearance.

**Figure 5:**
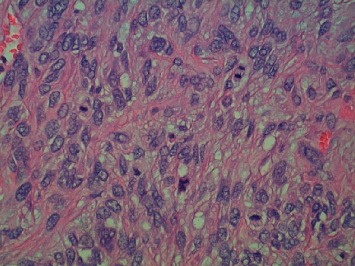
Hematoxylin and Eosin, high-power 500x view of solitary fibrous tumor. Four mitotic figures identified within a single high-power field.

The pathology of the right lower lobe tumor was a SFT. This tumor showed a fascicular pattern of spindled cells with elongated nuclei (see Figure 4). The mitotic rate was significantly elevated at 13 mitotic figures per 10 high-power fields (see Figure 5). Multiple fields of necrosis were also identified. Immunohistochemical stains were performed, which revealed reactivity for CD34 and Bcl-2. The tumor was negative for pancytokeratin. This tumor did not extend beyond the lung parenchyma. A routine sampling of the right lower paratracheal (4R), subcarinal (7), paraesophageal (8), and right hilar (10R) lymph nodes was negative for metastatic disease from either tumor. The surgical margins were negative for tumor in both cases. Thus, no additional therapy was recommended at that time. He was followed with clinical visits and imaging studies.

**Figure 6:**
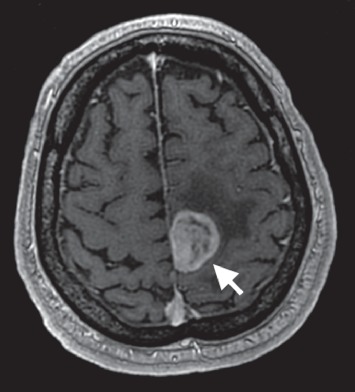
T1 postgadolinium MRI brain with axial view. This reveals a peripherally enhancing and centrally necrotic appearing mass in the left frontoparietal lobe with surrounding edema.

**Figure 7:**
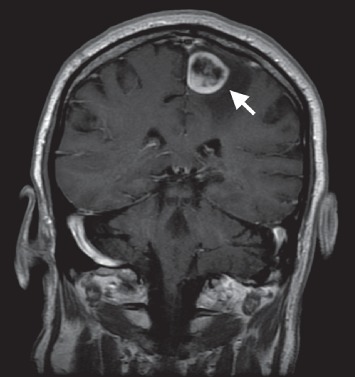
T1 postgadolinium MRI brain with coronal view. This reveals a peripherally enhancing and centrally necrotic appearing mass in the left frontoparietal lobe with surrounding edema.

The patient did well over the next 15 months. However, a restaging CT chest with contrast showed multiple new pulmonary tumors. Shortly after this CT scan was performed, he presented with mild right leg weakness. MRI of the brain showed a solitary 2.5 cm enhancing mass of the left posterior frontal lobe, just anterior to the motor cortex, with extensive surrounding edema (see Figures 6 and 7). The timing of his new symptoms and the imaging findings were consistent with a metastatic brain lesion.

**Figure 8:**
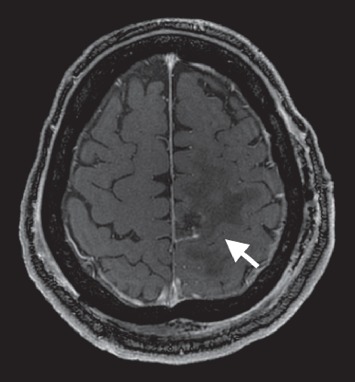
Postresection, T1 postgadolinium MRI brain with axial view. This reveals postsurgical changes after a gross total resection of the left frontoparietal mass with residual edema in the tumor bed.

**Figure 9:**
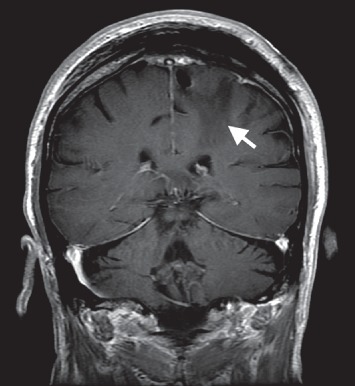
Postresection, T1 postgadolinium MRI brain with coronal view. This reveals postsurgical changes after a gross total resection of the left frontoparietal mass with residual edema in the tumor bed.

**Figure 10:**
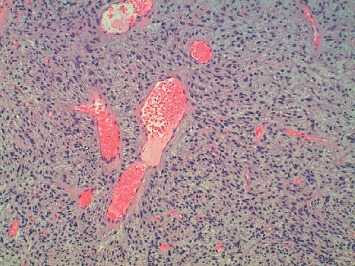
Hematoxylin and Eosin, low-power 100x view of metastatic solitary fibrous tumor. The tumor again shows a mixture of small slit-like vessels and large ectatic vessels with a characteristic “staghorn” appearance.

**Figure 11:**
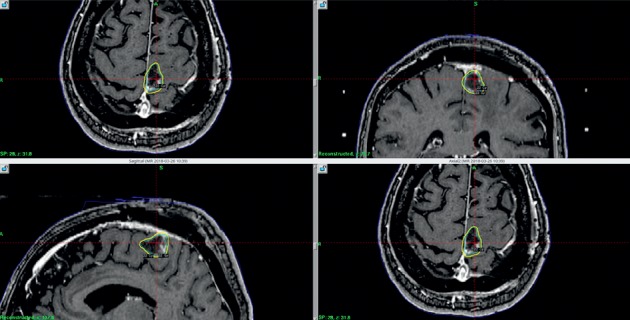
Multiple views of the Gamma Knife treatment plan with 22 Gy prescribed to the periphery of the tumor cavity, and this correlates with the 50% isodose line (dose representations are overlayed on a brain MRI from the day of treatment).

The patient underwent left frontal craniotomy with gross total tumor resection, as shown on postoperative MRI (see Figures 8 and 9) without complications. The brain resection pathology revealed a malignant solitary fibrous tumor. It appeared similar to the SFT resected from the lung. It was comprised of spindled cells with storiform and haphazard arrangement. The tumor matrix was predominantly myxoid, and the cells were fed by a rich vascular network of small slit-like vessels and large ectatic branching vessels (see Figure 10). The tumor showed immunoreactivity for CD34, Bcl-2, and STAT-6. The Ki-67 proliferation index approached 30%. His case was discussed at the regional multidisciplinary brain tumor board where the consensus recommends radiosurgery. He underwent single fraction Gamma Knife radiosurgery to the tumor cavity with a prescription dose of 22 Gy to the 50% isodose line (see Figure 11). His weakness subsequently resolved.

The new right upper lobe lung metastasis was biopsied and found to be metastatic adenocarcinoma. He was prescribed systemic treatment with pembrolizumab, carboplatin, and Alimta and tolerated systemic therapy well but does continue to have generalized weakness, generalized dyspnea on exertion, and a chronic dry cough. At the time of this report, it has been 16 months since the Gamma Knife treatment and he has not developed subsequent recurrence of brain metastasis. He continues to receive treatment with pembrolizumab.

## 3. Discussion

This is an unusual case of routine adenocarcinoma of the lung, but also SFT of the lung. Furthermore, the patient developed a symptomatic solitary brain metastasis from the SFT, not the lung adenocarcinoma, even more unusual and unexpected.

SFTs are rare mesenchymal neoplasms that account for less than 2% of all reported soft tissue tumors [1], first reported in the literature by Wagner in 1870 [5]. SFTs were originally thought to only arise in the thoracic cavity, but it is now known that while most SFTs are intrathoracic and usually from the pleura, these tumors are frequently found in various locations throughout the body [6, 7]. Peritoneal cavities, usually the retroperitoneum or pelvic soft tissue, are the primary site of origin in 30% of SFTs [8, 9]. 20% occur in the head and neck, the meninges, or extracranial locations [10–12]. These can include the sinonasal tract, oral cavity, and orbit [13–15]. There have also been cases of SFT arising from the extremities and bone as well as very few reported from the abdominal wall [16–18]. SFTs are most commonly benign, but 10–20% of cases have been reported to be malignant. SFTs usually occur in middle-aged patients and equally in men and women [2]. The most common sites for metastasis are the lungs (61%), pleura (49%), liver (41%), bones (41%), and peritoneum (41%) [3]. SFT has a low reported incidence of metastasis to the brain, especially low when compared to lung adenocarcinoma [4].

SFTs are usually considered chemoresistant; therefore, localized treatments with surgery or radiation therapy are the most common modalities used. Initially, surgery is most effective, and tumor removal with negative margins of 1 to 2 cm, if possible, is recommended. Complete surgical resection of localized lesions is thought to be important for optimal outcomes [3]. Though the literature is inconclusive about the most effective use, radiotherapy is a good adjuvant treatment before or after surgery in more complex conditions, such as in the case of an incomplete resection or if the tumor is inoperable. We have previously published clinical outcomes after radiosurgery or whole brain radiation for brain metastasis of various histologic types [19, 20]. Radiation therapy is especially utilized in cases with malignant SFT, narrow margins, tumor size greater than 10 cm, and fast-growing tumors [2]. For example, in an analysis of 102 SFT cases, Krengli et al. reported that local control was higher in patients who were treated with surgery plus postoperative radiation therapy when compared to surgery alone [21]. If the tumor has metastasized, adjuvant radiation therapy and sometimes chemotherapy are also used. However, Duranti et al. summarized their treatment of 337 patients with localized thoracic soft tissue sarcoma, where 51% received adjuvant radiotherapy and 41% received postoperative chemotherapy, and concluded the patients who received adjuvant radiotherapy presented with better local control than those who received adjuvant chemotherapy alone [22].

When compared to brain metastasis from adenocarcinoma, brain metastasis from SFT is significantly less common. In a study of 16,210 patients with brain metastasis, the authors reported that brain metastasis from lung cancers (19.9%) occurs more frequently when compared to other types of cancer, such as melanoma (6.9%), renal (6.5%), breast (5.1%), and colorectal (1.8%) [4]. Another study reported similar outcomes with a higher incidence of brain metastasis in lung cancer (16.3%) when compared to renal carcinoma (9.8%), melanoma (7.4%), breast carcinoma (5.0%), and colorectal carcinoma (1.2%) [23]. Of lung cancers, a study of 485 cases of patients with lung cancer by Villano et al. reported that the highest occurrence of brain metastasis was from adenocarcinomas [24]. In addition, in a 2018 study of 373 patients with adenocarcinoma of the lung, brain metastasis was reported to be one of the most common sites of distant metastasis and have a significantly higher rate of brain metastasis than other types of non-small-cell lung cancer [25]. The relatively high metastatic rate to the brain from adenocarcinoma of the lung is historically documented in that a study published in 1988 reported brain metastasis in 28% of their 259 patients with adenocarcinoma of the lung [26].

SFT has a much lower incidence of brain metastasis than adenocarcinoma. In a study of 139 patients with SFT (49 of which had metastases), O'Neill et al. reported that the most common sites of metastasis were the lungs (61%), pleura (49%), liver (41%), bones (41%), and peritoneum (41%). Only 8 of their 49 patients with metastases had brain metastases. The authors report that 5 patients had multiple brain metastases while 3 patients had single brain metastases. Six of the patients with brain metastases had tumors located extraaxially, 1 had extraaxially, and 1 had both extraaxially and intraaxially. The metastases were hypervascular in half of the patients, and the largest diameter was 1.4 cm (range, 0.5–2.8 cm). O'Neill et al. stated that brain metastases were most commonly from head and neck primary SFT, as seen in 5 patients, but also arose from 2 patients with thoracic SFT and 1 with abdominal SFT [3]. Very few brain metastases from SFT have been reported in the literature.

Surgical excision generally yields a good prognosis for most SFT patients, as seen in a study by Gold et al. of 75 patients with SFTs in a wide range of anatomic sites. The authors reported that the factors predicting worse prognosis include positive surgical margins, large tumor size greater than 10 cm, and the presence of a histologically malignant component [1]. Another study by van Houdt et al. similarly found that the variables most correlated with local recurrence and worse prognosis overall included positive resection margin, tumor size greater than 10 cm, and high mitosis rate. Five years after surgical resection of the 81 patients they followed, overall survival was 84%, local recurrence rate was 29%, and metastasis rate was 34% [27]. Franzen et al. also emphasized the importance of a high mitotic index in recurrence and worse prognosis after following 42 SFT patients [28]. A study by Tapias et al. reviews 59 patients with surgical treatment of SFT of the pleura (SFTP), one of the most common anatomical locations of SFT, to propose a score that can help predict recurrence of SFTP and guide postoperative surveillance. They report that their scoring system was better at predicting malignant behavior and recurrence than some other scores, such as England's criteria or de Perrot staging. Their predictive score focuses on factors including mitotic activity, necrosis, sessile morphology, presence of hypercellularity, and size [29]. However, Franzen et al. in their study found the factors of tumor necrosis and sessile morphology to be statistically insignificant [28].

Localized treatment for primary tumor typically consists of surgery with or without radiotherapy for SFT. SFT can be chemoresistant, or chemotherapy may have inconsistent effects in some patients, but a study at MD Anderson concludes that conventional chemotherapy, such as doxorubicin-based therapy, gemcitabine-based therapy, and paclitaxel, can be helpful in controlling or stabilizing locally advanced and metastatic SFT [30]. Another study at MD Anderson reported that combination therapy with temozolomide and bevacizumab was well tolerated and almost as beneficial as surgery [31]. However, surgical excision still remains the treatment of choice. van Houdt et al. reported the factor most significantly correlated with local recurrence is the positive resection margin with a hazard ratio of 4.8 (confidence interval 95%) [27]. Compared to patients with a negative margin, patients with a positive resection margin have a statistically significant higher risk of local recurrence. In a study of 220 cases reported of intracranial and intraspinal SFT, Bisceglia et al. recommends surgery as the main treatment as well as stereotactic and external beam radiotherapy for unresectable tumors and some cases of postsurgical tumor remnants [32]. Adjuvant radiotherapy is also often used for cases with extensive external invasion. In a very recent study, Bishop et al. reviewed 31 patients treated for SFT in multiple anatomic sites using both surgery and radiation therapy and reported 5-year rates of local control, overall survival, and distant metastatic-free survival as 100%, 95%, and 92%, respectively [33]. In another very recent study including 14 SFT patients treated with postoperative radiation therapy, Jia et al. also recommends adjuvant radiotherapy to lower recurrence rate [34].

## 4. Conclusion

We present a unique case of a patient with simultaneously diagnosed solitary fibrous tumor of the right lung and adenocarcinoma of the right lung, and who was later found to have a solitary fibrous tumor metastasis to the brain with a single left frontal lobe lesion. This patient received neurosurgical resection for the brain metastasis followed by Gamma Knife treatment to the surgical cavity. At this time, there are no other reported cases with this specific course of treatment for the single brain metastasis from solitary fibrous tumor pathology. Future research is still needed for this rare malignancy and clinical presentation.
